# Supplementary material for: Developmental trajectory of voluntary alcohol consumption in adolescent mice using finite mixture modeling and Bayesian posterior probability analysis
Source: PLoS One. 2025 Apr 11;20(4):e0321506. doi: 10.1371/journal.pone.0321506 (PMC11990736; doi:10.1371/journal.pone.0321506)
Supplement: Attachment — Submitted filename: Response to Reviewers.docx [file pone.0321506.s001.docx]

Response to Reviewers

**Reviewer #1:**

The authors present a new and unbiased approach to assessing mammalian voluntary ethanol consumption that has revealed identifiable patterns amongst seemingly heterogeneous individual behaviors. The application of finite fixture modeling to these data appears to be statistically appropriate and consistent with other published work utilizing this approach to assess variability in rodent behavior; however, I must include the caveat that I do not have expert-level knowledge of this modeling approach. Regarding the methods and conclusions, I cannot identify any major methodological problems with these studies; the paradigm used is well-established and appears to have been carried out with care and the use of appropriate controls, and the finding that the majority of animals do not escalate their ethanol intake when given unlimited access in a two-bottle choice procedure is consistent with the findings of many others. This study adds to our understanding of ethanol self-administration by identifying two distinct drinking trajectories amongst the minority of mice that do increase their ethanol intake over time, and future studies could apply this approach to determine whether their are behavioral or physiological differences in the "early" vs "late" drinkers, either at baseline or following adolescent ethanol access.

I do have two comments, one minor and one of moderate importance. Firstly, in Figure 4, food intake is only reported through day 38; given that Group 1 mice do not begin to escalate their ethanol intake until sometime after day 34, it could be that food intake is altered in this group once their ethanol self-administration increases. Did the authors measure food intake in these mice after day 41? Could their food intake have changed as their consumption increased?

Response: The reviewer’s point is well-taken. Unfortunately, we only had food intake data recorded for the first seven weeks, as depicted in Figure 4. To highlight this issue of potential concern, we both indicated it in the Results section, and also included the following paragraph in the Discussion section, “Based on the 3-group membership assignments, the body weight of the three groups of mice were comparable throughout the duration of the study (Fig 3). The three groups of mice also displayed comparable levels of food intake for the duration that food intake was measured (seven weeks from the beginning of the study, see Fig 4). It should be pointed out that the increase of alcohol intake of Group 1 started around the time of seven weeks, which may have an effect on food intake. We did notice that, however, for the high alcohol consumption of Group 3, their food intake levels were comparable to that of the other groups during the first seven weeks of the study, despite Group 3 mice consuming more alcohol during this period.”

My minor comment is that in section 2.3 there appears to be an accidental insertion of a partial citation (Gordon & Axelrod).

Response: We thank the reviewer for the astute observation of the error, which has been corrected in the revised submission.

**Reviewer #2:**

I find this study by Yu and colleagues to be genuinely thoughtful and carefully executed. I only have a small number of comments to help bolster the impact of the results and meaning of the data in the context of alcohol drinking studies using various approaches in mice.

1. In the Discussion, I suggest including a note about the very first significantly notable difference that emerges between the groups of mice indicated by the modeling approach. Is there a meaningful or sensitive way to predict the onset of those individuals most vulnerable to patterns of high intake? Would that ability to detect differences between vulnerable or resilient populations be valuable to future characterizations of molecular and cellular endpoints in adolescent mice?

Response: We thank the reviewer’s comments. Figure 2 has been modified to indicate the specific time points where significant differences were detected. Also, a note is included in the Discussion section concerning the first significantly notable difference. We particularly appreciate the reviewer’s insight regarding detecting early differences for future characterization of molecular and cellular endpoints in adolescent mice. Indeed, we are currently conducting a study to use machine learning algorithms for early prediction of alcohol consumption behavior, so that mice of each alcohol consumption type could be identified early on, to allow molecular profiling of their brain gene expression patterns. We hope to complete this study soon, and to submit a manuscript as a follow-up to the current one.

2. Also with regards to the Discussion more detail should be added about: (1.) the levels of alcohol intake, including a note about blood alcohol levels across the three groups of mice derived from the modeling approach, and (2.) how the levels (g/kg/day) of drinking in this study compare to levels consumed in similar and alternative/binge drinking studies (e.g., Matson and Grahame 2011-Addiction Biology; Thiele and Navarro 2015-Alcohol).

Response: We very much appreciate the reviewer’s suggestion, and the following has been included in the Discussion section, “As outbred mice, the high levels of alcohol consumption achieved by both Group 1 and Group 3 are comparable to several of the inbred mouse strains (Blednov et al., 2010), although still below those by C57BL/6 (Blednov et al. 2010; Matson and Grahame 2013; Thiele and Navarro 2014), as C57BL/6 mice are known to show higher ethanol intake than many other mouse strains (Belknap et al. 1993).”

3. In the Method Section, Section 2.2, please provide more details about the when the mice in this study received the two different concentrations of alcohol (i.e., 5% and 10%) and the frequency of each concentration's presentation over the course of alcohol access.

Response: We thank the reviewer for the suggestion, and the specifics were provided in the Method Section 2.2. Further, the following was included in the Discussion Section, “The voluntary alcohol consumption paradigm in our study compared two regimens of alcohol solution: 5% (mouse 1-18) and 10% (mouse 19-35). As shown in Table 3, it appears that there was no difference between the two regimens – half of the mice in Group 1 (late drinkers) were from the 5% regimen (mouse 1 and 18), and the other half were from the 10% regimen (mouse 29 and 31); similarly, Group 3 (early drinkers) membership was also relatively evenly distributed – three mice were from the 5% regimen (mouse 7, 13 and 14), and two were from the 10% regimen (mouse 24 and 30). This suggests that regardless of the alcohol concentration of the regimens, mice in a group consumed similar amounts of alcohol (as reported in g/kg/day, see Fig. 2).”
